# The long non-coding RNA LINC01089 facilitates prostate cancer progression

**DOI:** 10.1016/j.bbadva.2026.100205

**Published:** 2026-10-14

**Authors:** Sandra Vega Neira, Damu Tang

**Affiliations:** a Department of Medicine, McMaster University, Hamilton, ON, L8S 4K1, Canada; b The Research Institute of St Joe’s Hamilton, St Joseph’s Hospital, Hamilton, ON, L8N 4A6, Canada

**Keywords:** LINC01089, LncRNA, Prostate cancer, Biochemical recurrence, Xenograft tumors

## Abstract

**Background:**

Evidence suggests LINC01809 being involved in prostate cancer (PC). We studied LINC01089 functionality, the underlying mechanisms, and translational potential in PC.

**Methods:**

Multiple PC cohorts (local, TCGA, and others) were utilized. Human (DU145 and LNCaP) and murine (TRAMP-C2) PC cells were used to investigate LINC01089-derived oncogenic potential in vitro and in vivo.

**Results:**

LINC01089 upregulation occurred in PC, Gleason score 9–10 tumors and metastasis. LINC01089 stratified PC recurrence. Plasma collected from patients (n = 235) prior to treatment contained elevated LINC01089, which was further increased in higher Gleason PCs and modestly correlated with PSA score. LINC01089-positively correlated genes contained a 15-gene panel (Sig15) and were dominantly enriched in RNA splicing in multiple PC datasets, with three genes (DDX17, LUC7L3, and HNRNPH1/Overlap3) conserved. Overlap3 and Sig15 effectively stratified the recurrence risk of PC and poor prognosis across 9 other cancer types, and captured the tumors' RNA splicing property, a major mechanism contributing to androgen receptor (AR) variants. Ectopic LINC01089 enhanced DU145, LNCaP, and TRAMP-C2 PC cell proliferation, colony formation, and migration, and conferred LNCaP and TRAMP-C2 cell proliferation under androgen deprivation, along with AR upregulation in the nucleus. In C57BL/6 mice, TRAMP-C2 LINC01089 allografts grew faster and expressed increased nuclear AR, Ki67, and AKT activation. TRAMP-C2 LINC01089 allografts expressed higher levels of Overlap3 and some Sig15 genes.

**Conclusion:**

LINC01089 facilitates PC. Overlap3 and Sig15 are novel prognostic biomarkers reflecting cancer-associated RNA splicing. Plasma LINC01089 displays PC diagnostic potential.

## Highlights

• LINC01089 is increased in prostate cancer (PC), metastasis and predicts PC relapse.
• LINC01089-derived gene panels stratify poor overall survival across 9 cancer types.
• Plasma LINC01089 possesses translational applications in PC diagnosis.
• LINC01089 promotes PC cell proliferation and resistance to androgen withdrawal.
• LINC01089 enhances PC allograft growth with increases in androgen receptor expression.

## 1 Introduction

Prostate cancer (PC) is a leading cause of cancer-related deaths in men [1,2]. The disease at early stages is treated with radical prostatectomy and/or in combination with radiotherapy [3]. Approximately 30% of patients will experience relapses and progress into an aggressive form with high risk of metastasis [4,5]. Abnormal androgen receptor (AR) signaling is the major driver of PC oncogenesis and progression [6]. Metastatic PCs are standardly treated with androgen-deprivation therapy (ADT). While ADT offers effective control in the majority of patients, resistance in the form of castration-resistant prostate cancer (CRPC) commonly develops, driven largely by persistent AR signaling [6,7]. PC pathogenesis, progression, and resistance to therapy are affected by unique gene expression induced by treatment and genomic instability including aneuploidy [8,9], leading to alterations in multiple aspects of PC, including immunity and other components of the tumor microenvironment (TME) [8,10]. Despite extensive investigation into the mechanisms underlying PC oncogenesis, progression, and CRPC development, the current understanding of PC etiology remains incomplete.

Long non-coding RNAs (lncRNAs) are important regulators of oncogenesis [11,12]. In PC, PCA3 (prostate cancer antigen 3) is the most well-studied lncRNA and the first FDA-approved urine-based diagnostic biomarker for men with elevated PSA serum levels but negative biopsy results [2,13]. Additionally, evidence suggests the lncRNA LINC01089 is relevant to PC; LINC01089 is a component gene of a 27-gene signature (Sig27) panel of PC, which effectively predicts PC relapse [14]. The *LINC01089*/*LOC338799* gene is located on chromosome 12q24.31, acting predominantly as a decoy due to its capacity to interact with multiple DNA and RNA molecules [15]. Furthermore, LINC01089 plays a critical role in oncogenesis in a cancer-dependent manner; LINC01089 facilitates migration and invasion of hepatocellular carcinoma [16], whereas its downregulation enhances proliferation, migration and invasion in breast [17] and gastric cancers [18]. Nonetheless, the impact of LINC01089 on PC remains unknown.

We provided the first evidence for LINC01089 promoting PC. Clinically, LINC01089 upregulation occurs in PC following PC progression (higher Gleason score, recurrence, and metastasis) and in plasma, suggesting LINC01089’s diagnostic potential. This possibility is further supported by its strong correlation with serum PCA3, an FDA-approved auxiliary diagnostic biomarker of PC. Furthermore, from the LINC01089 network, Sig15 and Overlap3 were constructed. Both stratify PC recurrence in two independent datasets and show poor overall survival across multiple cancer types. Functionally, LINC01089 stimulates PC cell proliferation, migration, and colony formation, and sustains proliferation under androgen deprivation in vitro. In C57BL/6 mice, LINC01089 enhances TRAMP-C2 tumor growth; of relevance, TRAMP-C2 LINC01089 tumors capture the features of Sig15 and Overlap3. Taken together, we provide the first demonstration of LINC01089 as a novel PC promoter using multiple systems. This study has clear clinical relevance with respect to PC diagnosis and risk stratification.

## 2 Materials and methods

### 2.1 Clinical samples

Prostate cancer tissues were collected from Hamilton Health Sciences and approved by the Research Ethics Board (REB 11,114). Plasma samples were from the Ontario Tumor Bank (OTB), which is supported by the Ontario Institute for Cancer Research through funding provided by the Government of Ontario. Inclusion criteria: Patients diagnosed with primary PC, clinical data available, and sample collected prior to treatment.

Control plasma samples were from healthy individuals from the American Association for Clinical Chemistry (AACC), who were screened for diabetes, high blood pressure, renal disease, heart disease, deep vein thrombosis, cancer and any treatment.

### 2.2 Plasma collection and processing

According to the Standard Operating Procedure (SOP) of the OTB, whole blood was collected into a standard lavender EDTA vial and fractionated by centrifugation within 24 h. Plasma samples were aliquoted (1mL/vial) and cryopreserved in liquid nitrogen.

At the lab, the samples were stored at −80 °C upon arrival. Prior to RNA extraction, 800 µL of TRIzol reagent (Invitrogen) was added to 200 µL of plasma per sample to preserve the RNA and stored at −20 °C. RNA extraction was performed within 5 days. RNA concentration and purity were measured using a NanoDrop. RNA samples with an A260/280 and A260/230 ratio between 1.80 and 2.20 were used for further analysis. Hemolysis assessment was performed by calculating the A414/375 index. Samples with a hemolysis index >0.25 were considered hemolyzed and were excluded from the study.

### 2.3 PC cell lines

DU145 and LNCaP cells were obtained from the American Type Culture Collection (ATCC). TRAMP-C2 cells were obtained from Dr. Jehonathan H. Pinthus. All three cell lines were authenticated. DU145 and LNCaP cells were cultured in MEM and RPMI-1640 media. TRAMP-C2 cells were cultured in DMEM media. Mycoplasma contamination was routinely tested with a PCR kit (Abm, Cat#: G238).

### 2.4 Real-time PCR quantification

RNA was isolated using TRIzol (Invitrogen) following the manufacturer's procedure; cDNA was synthesized using Superscript IV (Thermo Fisher Scientific). Real-time PCR was performed on the QuantStudio 3 PCR system using Power SYBR Green PCR Master Mix (Thermo Fisher Scientific) in triplicate. The 2^−ΔΔCt^ method was used to calculate gene expression changes, with actin as the endogenous control. The primers used are listed in the supplementary Table S1. Intra-assay coefficient of variation (CV) was calculated as CV (%) = (SD/mean Cq) × 100, in which Cq (quantification cycle) correspond to the cycle number where the fluorescence signal crossed the preset threshold. A CV <2% reflects good reproducibility [19].

### 2.5 Cell transfection

p.LV-LINC01089 (VectorBuilder) and p.LV-empty vector (EV) (Addgene) were utilized to generate LINC01089 and EV lentivirus in 293T cells along with VSV-G and GP. DU145, LNCaP and TRAMP-C2 cells were then infected and selected with puromycin (1µg/mL, Sigma).

### 2.6 Cell proliferation and colony formation assay

Cell proliferation assays were performed in 6-well culture plates by seeding 10^4^ cells/well and counting cells every 2 days. Colony formation assays were performed by seeding cells in 6-well plates at the indicated density; colonies were stained with crystal violet (0.5%) and quantified. Assays were performed three times, either with complete culture media or androgen-free media (phenol red - free media supplemented with 10% Charcoal-stripped serum).

### 2.7 Wound-healing assay

Cells were cultured in a 6-well plate until 90% confluency. A horizontal scratch was then produced using a pipette tip, floating cells were removed, and cell migration was monitored every 6 h. Images were taken using the microscope (Axiovert 200, Carl Zeiss); the wound width was calculated using ImageJ software.

### 2.8 Immunofluorescence staining

Cells were fixed with 4% paraformaldehyde for 15 min, permeabilized with 0.1% Triton X-100 for 10 min, blocked for 1 hour at room temperature with blocking buffer (3% BSA + 0.3% Triton X-100 in PBS), and incubated with anti-AR (1:600, Cell Signaling) at 4 °C overnight. After washing with PBS, cells were incubated with FITC-conjugated Donkey anti-Rabbit IgG (1:200, Jackson ImmunoResearch) for 1 hour at room temperature, washed twice with PBS, and mounted using VECTASHIELD mounting medium containing DAPI (VECTOR Lab Inc.). Images were taken with a fluorescence microscope (Axiovert 200, Carl Zeiss).

### 2.9 Western blot analysis

Cell lysate protein (50 μg) was separated on an SDS-PAGE gel, followed by a standard Western blot procedure. Signal was developed (ECL Western Blotting Kit, Amersham), and the image was captured on a ChemiDoc MP system (Bio-Rad). The primary antibody used was anti-AR (1:1000, Cell signaling), and the secondary antibody used was goat anti-mouse IgG HRP-conjugate (Bio-Rad, 1:3000).

### 2.10 Generation of tumor allograft

TRAMP-C2 EV and LINC01089 cells (2 × 10^6^) were mixed in 0.1 mL of PBS and injected subcutaneously into 6–8-week-old C57BL/6 mice (eight mice per group). Tumor growth was monitored through palpation. Tumor size was measured using calipers and calculated based on the formula V=L×W2×0.52. Mice were euthanized when the tumour reached 1000 mm^3^ in volume. All the animal work was performed according to the protocols approved by McMaster University Animal Research Ethics Board (AUP # 2404).

### 2.11 Immunohistochemistry (IHC)

Slides were deparaffinized in xylene and cleared in 100%, 90% and 70% ethanol series. Antigens were retrieved in sodium citrate buffer (pH 6.0). Slides were blocked in PBS containing 1% BSA and 10% normal goat serum (Vector Laboratories) for 1 hour and incubated with either anti-Ki67 (1:200, Santa Cruz) or anti-AR (1:200, Santa Cruz) overnight at 4 °C. Secondary antibody (biotinylated goat anti-rabbit IgG) and Vector ABC reagent (Vector Laboratories) were added. A chromogenic reaction was performed with diaminobenzidine (Vector Laboratories), and counterstained with hematoxylin (Sigma-Aldrich). Images were acquired using a Nikon Eclipse Ti2 microscope, and DeepLIIF software was used to quantify positive cell staining based on the intensity and nuclear segmentation of at least 200 cells.

### 2.12 Statistical analysis

Statistical analysis was performed using R. Data are represented as mean ± SD (standard deviation) from three independent experiments. Student's *t*-test or one-way ANOVA was used to determine the significance between individual groups, followed by the Tukey-HSD test for comparison between multiple mean groups as indicated. Pearson correlation was used to evaluate the relationship between gene expression and clinical data. Results were considered significant with *p* < 0.05.

## 3 Results

### 3.1 LINC01089 facilitates prostate cancer

LINC01089 is a component gene of Sig27, a PC biomarker [14]; however, its role in PC is unclear. To study the relevance of LINC01089 in PC, we observed higher LINC01089 expression in brain metastasis-derived DU145 and bone metastasis-derived PC3 cells compared to lymph node metastasis-derived LNCaP cells (Fig. 1A). In our PC (n = 26) cohort containing eleven pairs of prostate tissues and adjacent non-tumor tissues, low-grade PC (Grade Group 1, GS5–6, n = 4), and advanced PC (Grade Group 5, GS9/10, n = 7) cases (Supplementary Table S2), we detected LINC01089 upregulation in PC tissue compared to adjacent non-tumor tissue (Fig. 1B) and in advanced PC over low-grade PCs (Fig. 1C). We further confirmed LINC01089 upregulation in PC (Fig. 1D), tumors progressing to relapse (biochemical recurrence - BCR) and lymph node metastasis (Fig. 1E, F), and distant metastasis (Fig. 1G). Moreover, LINC01089 upregulation is associated with increased risk of PC recurrence (progression-free survival/PFS) (Fig. 1H). To evaluate LINC01089′s relationship with PC pathogenesis, we benchmarked it against PCA3, a well-studied lncRNA upregulated in PC and an auxiliary biomarker in PC diagnosis [20]. PCA3 expression was significantly increased in PC in the TCGA dataset (Supplementary Fig. S1A), but its expression was not upregulated in advanced PCs in our dataset (Supplementary Fig. S1B), likely due to the small sample size. While PCA3 stratified PC recurrence risk (Supplementary Fig. S1C), its biomarker performance is less effective compared to LINC01089 (Supplementary Fig. S1D).

**Fig. 1:**
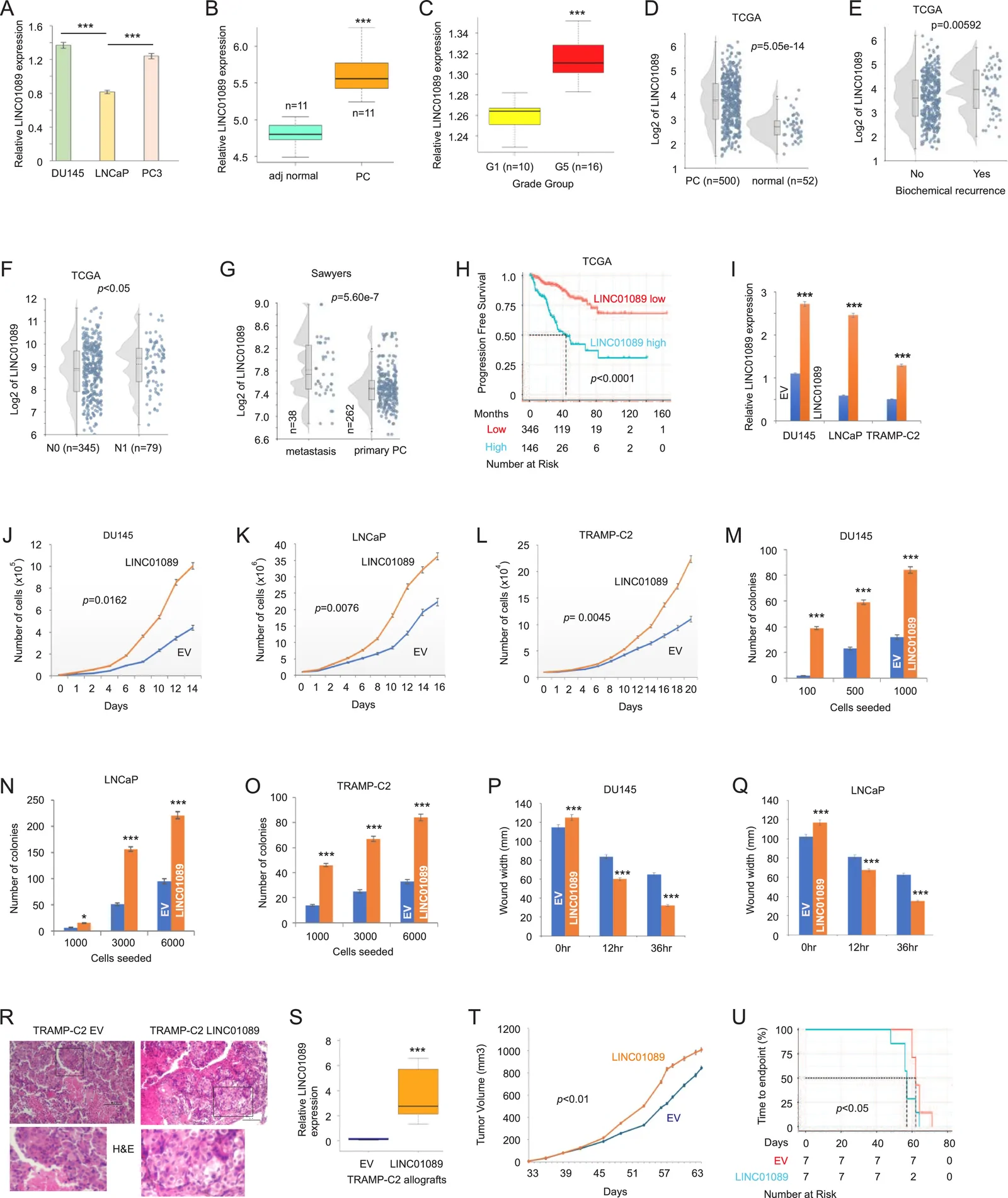
LINC01089 enhances PC pathogenesis. (A-C) Quantification of LINC01089 RNA expression by real-time qPCR in three human PC cell lines (A), 11 pairs of PC tissues and adjacent normal tissue (B), as well as in Grade group 1 (G1; Gleason score 5, 6) and group 5 (Gleason score 9, 10) PCs (C). The 2^−ΔCt^ method was used to calculate relative expression, which was normalized to actin. Mean±SD (Standard Deviation) are graphed. *** *p* < 0.001 by 2-tailed Student’s *t*-test. (D-G) LINC01089 expression in PCs vs normal prostate tissues (D), PCs with and without progression to 1) biochemical recurrence (E) and 2) lymph node metastasis (F), and primary vs metastatic PCs (G). The indicated datasets were used for the analysis on the R2: Genomics Analysis and Visualization Platform (http://r2.amc.nlhttp://r2platform.com). Statistical analyses were performed by the R2 Platform using one-way ANOVA. (H) LINC01089 Kaplan-Meier survival curves and log-rank test were carried out with the R survival package. Cut-off points were estimated using Maxstat. (I) LINC01089 expression in the indicated stable lines was determined using real-time qPCR. Relative LINC01089 expression mean±SD was graphed. *** *p* < 0.001 compared to the respective EV by 2-tailed Student’s *t*-test. (J, K, L) In a 6-well culture plate, the indicated stable lines were seeded at 10^4^ cells/well for DU145 and TRAMP-C2, and 10^6^ cells/well for LNCaP, followed by counting cells at the indicated time. Experiments were repeated three times. The area under the curve (AUC) associated with individual curves was used for statistical analysis by one-way ANOVA. (M, N, O) The indicated EV and LINC01089 stable lines were seeded in 6-well plates at the indicated density and cultured for 8 days till colonies formed, which were stained with 0.5% crystal violet. Typical images are presented in Supplementary Fig. S2A-C. Experiments were repeated three times; mean±SD are graphed. * *p* < 0.05, *** *p* < 0.001 by 2-tailed Student *t*-test. (P, Q) The indicated stable lines were seeded in a 6-well plate until reaching 90% confluency. A horizontal scratch was made, and migration was monitored every 6 h. Images were taken under the light microscope, and the wound width (mm) was measured using ImageJ software. Experiments were repeated three times. Quantifications were presented as mean±SD. * *p* < 0.05, *** *p* < 0.001 by 2-tailed Student *t*-test. (R) H&E staining of TRAMP-C2 tumor allograft. Typical images are shown, and a 2X enlargement of the marked zone. Scale bar: 100 µm. (S) LINC01089 RNA expression in TRAMP-C2 EV tumor (n = 7) and TRAMP-C2 LINC01089 allografts (n = 7). The 2^−ΔCt^ method was used to calculate relative LINC01089 expression, which was normalized to actin. Relative expression was graphed as mean±SD (Standard Deviation). *** *p* < 0.001 by 2-tailed Student’s *t*-test. (T) Tumor volume was measured in TRAMP-C2 EV (n = 7) and LINC01089 (n = 7) groups at the indicated time points. Growth curves are presented as mean±SD. Area under the curve (AUC) for each growth curve was calculated. Statistical analysis was performed using Student's *t*-test. (U) Time-to-volume point graph. Statistical analysis was performed using the Log-rank test.

To study the functional impact of LINC01089 on PC, we stably expressed LINC01089 in DU145, LNCaP, and TRAMP-C2 cells (Fig. 1I). Utilization of this strategy aligns with LINC01089 heterogenous expression with its expression at high levels in some PCs (Fig. 1D). Ectopic expression of LINC01089 elevated the proliferation rate of DU145, LNCaP, and TRAMP-C2 cells compared to the respective empty vector (EV) cells (Fig. 1J-L) and increase colony-forming ability (Fig. 1M-O; Supplementary Fig. S2A-C). Both DU145 LINC01089 and LNCaP LINC01089 cells closed the wound (gap) faster, suggesting an increase in cells’ motility ability (Fig. 1P, Q; Supplementary Fig. 2D, E), which may reflect the proliferation and migration properties of DU145 LINC01089 and LNCaP LINC01089 cells. Given that the wound healing assays were performed when cells reached 90% confluency (Supplementary Fig. 2D, E) and the time points included 12 h (Fig. 1P, Q), conditions utilized in our wound healing assay minimized cell proliferation, implying that the observed rapid gap closure in DU145 LINC01089 and LNCaP LINC01089 cells was likely attributed to enhanced migration ability. Additionally, cancer cells with elevated oncogenic potential, such as acquired EMT (epithelial-mesenchymal transition), can simultaneously display increased proliferation and migration capacities [21]. We thus performed a wound healing experiment without using mitomycin-C. We subsequently produced allografts in C57BL/6 mice using TRAMP-C2 EV and TRAMP-C2 LINC01089 cells (Fig. 1R) and confirmed elevated LINC01089 expression in TRAMP-C2 LINC01089 tumors (Fig. 1S). TRAMP-C2 LINC01089 tumors grew faster (Fig. 1T) and reached the volume endpoint in a shorter time (Fig. 1U). Collectively, these findings provide comprehensive evidence revealing that LINC01089 facilitates PC oncogenesis.

### 3.2 LINC01089 upregulates the nuclear content of androgen receptor (AR)

Given that LNCaP cells expressed the lowest level of endogenous LINC01089 among the three PC lines (Fig. 1A), LNCaP cells were further investigated for the impact of LINC01089 expression on PC progression. Additionally, LNCaP cells are androgen sensitive, providing a platform to evaluate the influence of LINC01089 on androgen receptor (AR) signaling, a key driver of PC cell proliferation [22,23], we observed significant nuclear enrichment of AR in LNCaP LINC01089 cells (Fig. 2A) with a modest upregulation of AR (Fig. 2B). The nuclear accumulation of AR strongly suggested its functional activation [6,24], indicating that LINC01089 may confer resistance to androgen withdrawal. Androgen withdrawal did not affect LINC01089 expression in LNCaP EV and LNCaP LINC01089 cells (Fig. 2C), suggesting LINC01089 is unlikely to be an AR target. In androgen-sensitive LNCaP and TRAMP-C2 cells, androgen deprivation significantly reduced the proliferation of both EV and LINC01089 cells (Fig. 2D, E); in comparison, the LINC01089 stable lines retained a proliferation advantage (Fig. 2D, E). While androgen deprivation decreased AR expression in both LNCaP EV and LNCaP LINC01089 cells (Fig. 2B), LNCaP LINC01089 cells expressed higher level of AR compared to EV (Fig. 2B), suggesting a role of AR in conferring LNCaP LINC01089 cells sustained proliferation under androgen-deprived conditions (Fig. 2D). Of importance, allografts produced by TRAMP-C2 LINC01089 cells contained a higher level of nuclear AR (Fig. 2F). Collectively, these in vitro and in vivo evidences imply that LINC01089 facilitates PC cell proliferation in the presence and absence of androgen, in part by enhancing AR signaling.

**Fig. 2:**
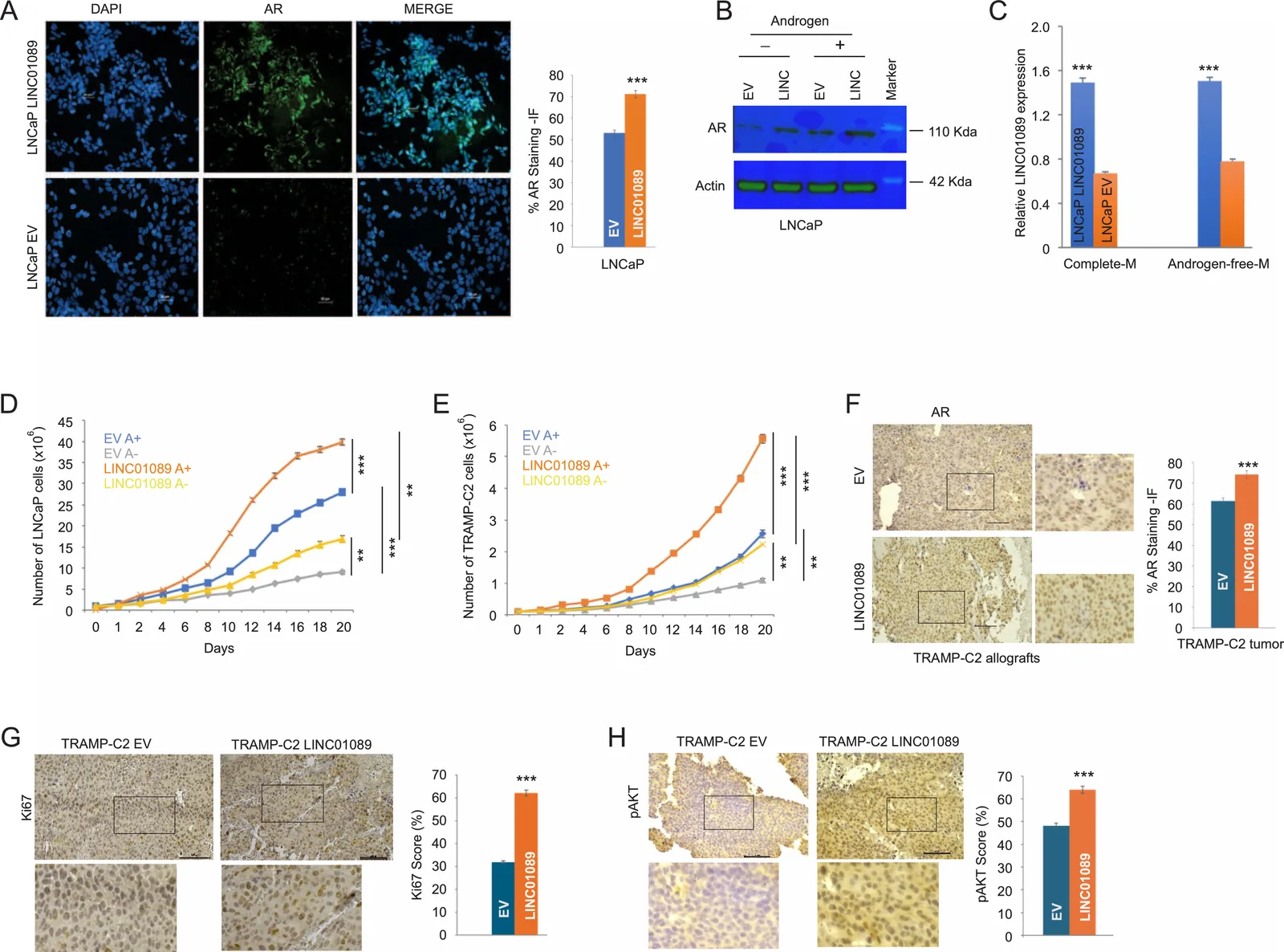
LINC01089 facilitates AR signaling. (A) Immunofluorescence (IF) staining for AR in LNCaP EV and LINC01089 cells using an anti-AR antibody. DAPI was used for counterstaining. Typical DAPI, FITC, and merged images are presented. Quantification of nuclear AR percentage is presented as mean±SD. *** *p* < 0.001 by 2-tailed Student *t*-test. (B) LNCaP EV and LINC01089 cells were cultured in complete media (+) and androgen-free media (-). Western blot was carried out using 50 µg total cell lysates to analyze AR and Actin expression. (C) LNCaP EV and LNCaP LINC01089 cells were cultured in complete or androgen-free media for 48 h, followed by real-time PCR analysis. Experiments were repeated three times. LINC01089 expression was normalized to Actin and presented as relative expression (means±SD). *** *p* < 0.001 compared to LNCaP EV cells by 2-tailed Student *t*-test. (D, E) The LNCaP cell lines were seeded at 10^6^/well in a 6-well plate; the TRAMP-C2 lines were seeded at 10^4^ cells/well. The cells were cultured in complete (A+) or androgen-free (A-) media. Cells were counted at the indicated time point. Quantifications of three independent experiments are presented as mean±SD. Area under the curve (AUC) for each growth curve was calculated. ** *p* < 0.01, ** *p* < 0.001 by 2-ways ANOVA. (F-G) Immunohistochemistry (IHC) staining of nuclear AR (F), Ki67 (G), and pAKT (H) in TRAM-C2 EV tumors (n = 7) and TRAM-C2 LINC01089 allografts (n = 7). Typical images are shown, and a 2X enlargement of the marked zone. Scale bar: 100 µm. Images were acquired using a Nikon Eclipse Ti2 microscope, and DeepLIIF (Deep Learning Inferred ImmunoFluorescence) software was used to quantify positive staining based on the intensity and cell segmentation. Quantifications are presented as mean ± SD. *** *p* < 0.001 by 2-tailed Student *t*-test.

TRAMP-C2 LINC01089 tumors expressed elevated levels of Ki-67 and AKT activation (phosphorylated AKT/pAKT) (Fig. 2G, H). Given the reciprocal relationship between AR and PI3K-AKT signaling in promoting PC progression [25,26], LINC01089 likely facilitates PC progression via utilization of both the AR and PI3K-AKT signaling.

### 3.3 Correlation between LINC01089 gene network and RNA splicing pathway

Given that oncogenic factors influence tumorigenesis via their molecular networks, we defined the LINC01089-associated network using two approaches. Genes positively correlated with LINC01089 expression (R ≥ 0.55, *p* < 0.0001) were derived from the PRAD-TCGA dataset on R2 genomics Analysis and Visualization Platform (https://hgserver1.amc.nl/cgi-bin/r2/main.cgi?open_page=login); the top two pathways enriched in these LINC01089 positively correlated genes were mRNA synthesis and RNA splicing regulation (Supplementary Fig. 3A). LINC01089 separates PCs into a high- and low-risk group of recurrence in the TCGA dataset (Fig. 1H); we obtained differentially expressed genes (DEGs; fold change≥|2|, *q* < 0.001) between both groups and demonstrated dominant enrichment in RNA splicing (Fig. 3A).

**Fig. 3:**
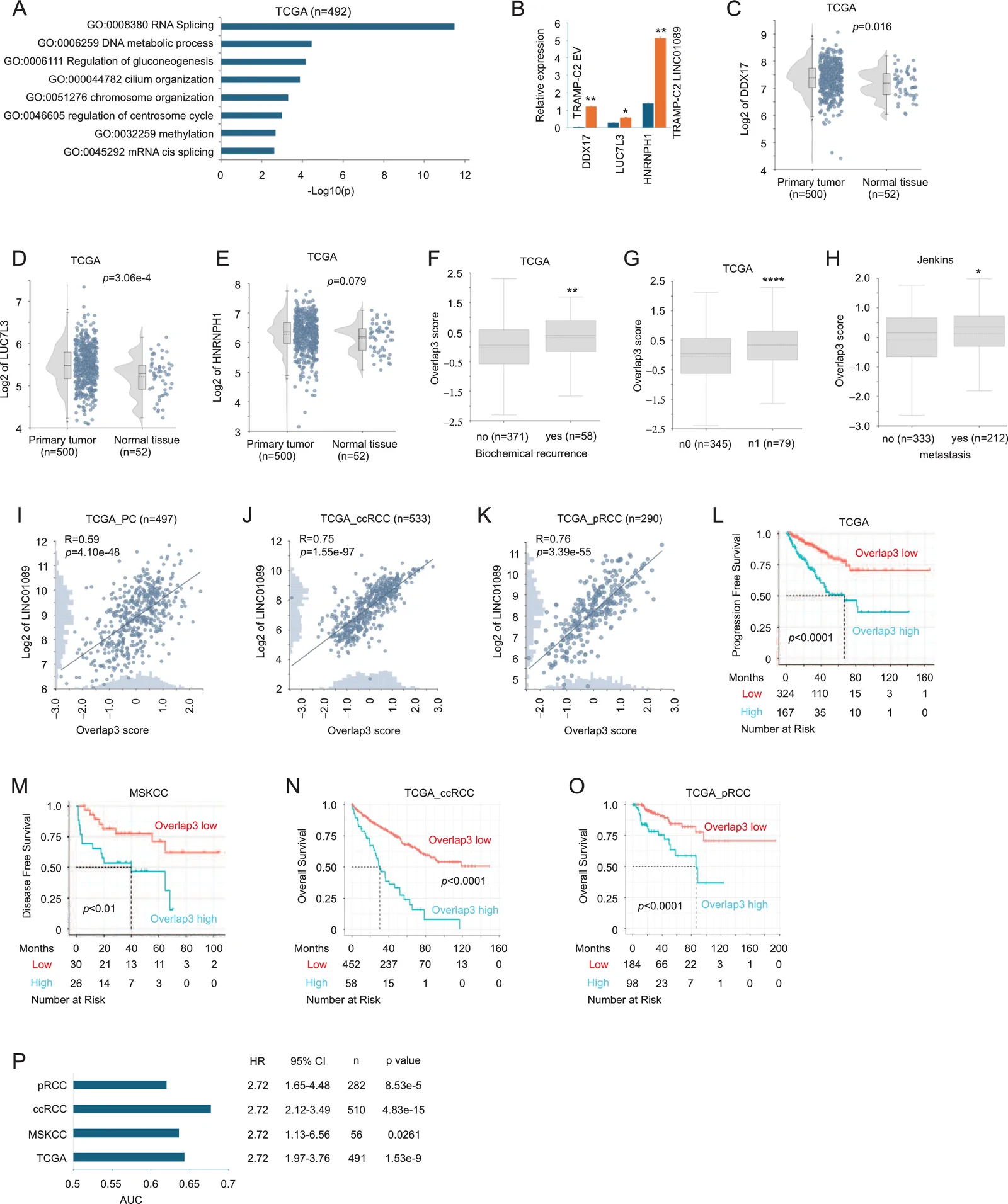
Association of LINC01089 with the RNA splicing pathway. (A) Pathway enrichment analysis of LINC01089-derived DEGs (n = 310, fold change≥|2|, *q* < 0.001) on the TCGA-PRAD (n = 492) dataset. Analysis was performed using Metascape (https://metascape.org/gp/index.html#/main/step1). (B) Relative gene expression of the indicated genes was performed by real-time qPCR in EV (n = 6) and LINC01089 (n = 6). Quantifications are presented as mean±SD. * *p* < 0.05, ** *p* < 0.01 by 2-tailed Student *t*-test. (C-E) DDX17, LUC7L3, and HNRNPH1 mRNA expression in primary PC tissues (n = 500) and normal prostate tissues (n = 50) using the TCGA PC dataset. (F-H) Overlap3 metagene expression in the indicated PC groups in the TCGA-PRAD (n = 552) and Jenkins (n = 545) datasets. (I-K) Pearson correlation analysis between LINC01089 and Overlap3 metagene expression in the indicated TCGA PC (I), TCGA ccRCC (J), and TCGA pRCC (K) datasets. Analyses were performed using R2: Genomics Analysis and Visualization Platform (http://r2.amc.nlhttp://r2platform.com). Statistical analyses were performed by the R2 platform using one-way ANOVA. (L-O) Overlap3 stratification for PC recurrence (L, M) and poor overall survival in ccRCC (N) and pRCC (O) in the indicated datasets. Cutoff points were estimated using Maxstat, and log-rank tests were carried out with the R survival package. (P) Overlap3 stratification effectiveness of PC recurrence risk in two PC datasets and inferior OS in ccRCC and pRCC by the ROC-AUC, and its estimated hazard ratio (HR) as indicated. Analysis was performed using the R Survival package.

We obtained LINC01089 positively correlated genes from the Jenkins (primary PC, n = 333; R ≥ 0.5; *p* < 0.05), Sawyers (metastasis, n = 38; R ≥ 0.7; *p* < 0.05), and Stampede (GS6–7 PC, n = 348; R ≥ 0.5; *p* < 0.05) datasets. An enrichment pathway analysis identified RNA splicing as a top pathway and revealed three conserved RNA splicing genes: DDX17 (Dead box Helicase 17), LUC7L3 (LUC7-like-3 pre-mRNA splicing factor) and HNRNPH1 (Nuclear ribonucleoprotein H1) (Supplementary Fig. 3B), collectively referred to as Overlap3. All three genes are involved in pre-mRNA processing and splicing [27]. These observations thus suggested a common correlation of LINC01089 with RNA splicing in PC. We further validated this by showing the upregulation of all three genes in TRAMP-C2 LINC01089 allografts compared to EV tumors (Fig. 3B), and in PCs over normal prostate tissues (Fig. 3C-E), with HNRNPH1 significance at *p* = 0.079 (Fig. 3E).

We further showed upregulation of the Overlap3 metagene in PCs progressing to relapse and lymph node metastasis (Fig. 3F, G), and distant metastasis (Fig. 3H). Overlap3 expression displayed a strong correlation with LINC01089 in the TCGA dataset (Fig. 3I); intriguingly, stronger correlations were detected in two major subtypes of renal cell carcinoma (RCC): clear cell RCC (ccRCC) and papillary RCC (pRCC) (Fig. 3J, K), revealing a general association of LINC01089 with Overlap3, likely reflecting their common relationship with RNA splicing. To study the biomarker potential of Overlap3, we computed its cohort-specific risk score for individual tumors as ∑(coef_i_ x gene_i_ expression)_n_, where n = 3 and individual Cox coefficients (coef) were obtained by multivariate Cox analysis. The cutoff point was estimated using Maximally Selected Rank Statistics. The Overlap3 risk score stratified recurrence risk in two independent PC datasets: the TCGA and MSKCC datasets (Fig. 3L, M), and poor overall survival (OS) in ccRCC and pRCC (Fig. 3N, O). The assessment appeared more effective, or at least comparable, in ccRCC compared to PC (Fig. 3P). Additionally, the cohort-specific Overlap3 risk score estimates the event hazard with a similar hazard ratio (HR) (Fig. 3P), implying that RNA splicing alterations captured by Overlap3 exert similar influences on the aggressiveness of PC, ccRCC, and pRCC. Of relevance, dysregulated RNA splicing is responsible for the generation of AR variant 7, which is commonly observed in CRPC tumors [[28], [29], [30]].

### 3.4 The biomarker potential of the LINC01089 network

The derivation of Overlap3 highlights the biomarker potential of the LINC01089 network. We further investigated LINC01089 DEGs (Supplementary Table 3) for assessing PC recurrence. A dataset was organized by retrieving these DEGs' expression data and relapse (biochemical recurrence) data from the TCGA dataset and randomized into Training (n = 253) and Testing (n = 239) subpopulations at a 6:4 ratio. Using the Training population, we carried out covariate selection (n = 310; Supplementary Table 3) for predicting recurrence risk using Elastic-net on the R glmnet package, obtaining a 15-gene panel (Sig15) (Supplementary Table 4). All 15 genes were upregulated in the high-risk group stratified by LINC01089 (Fig. 1H; Supplementary Table 4). Sig15 risk scores for individual tumors were calculated as ∑(coef_i_ x gene_i_ expression)_n,_ and the cutoff point was estimated. Sig15 risk score effectively stratifies recurrence risk in the Training cohort (Fig. 4A), as expected. We validated Sig15 in the Testing cohort using the coefficients derived from the Training cohort (Fig. 4B) and evaluated Sig15 biomarker potential in the full cohort again using the Training cohort-derived coefficients (Fig. 4C). Sig15 remained a predictor of PC recurrence after adjustment for age at diagnosis, T and N stage (Fig. 4D).

**Fig. 4:**
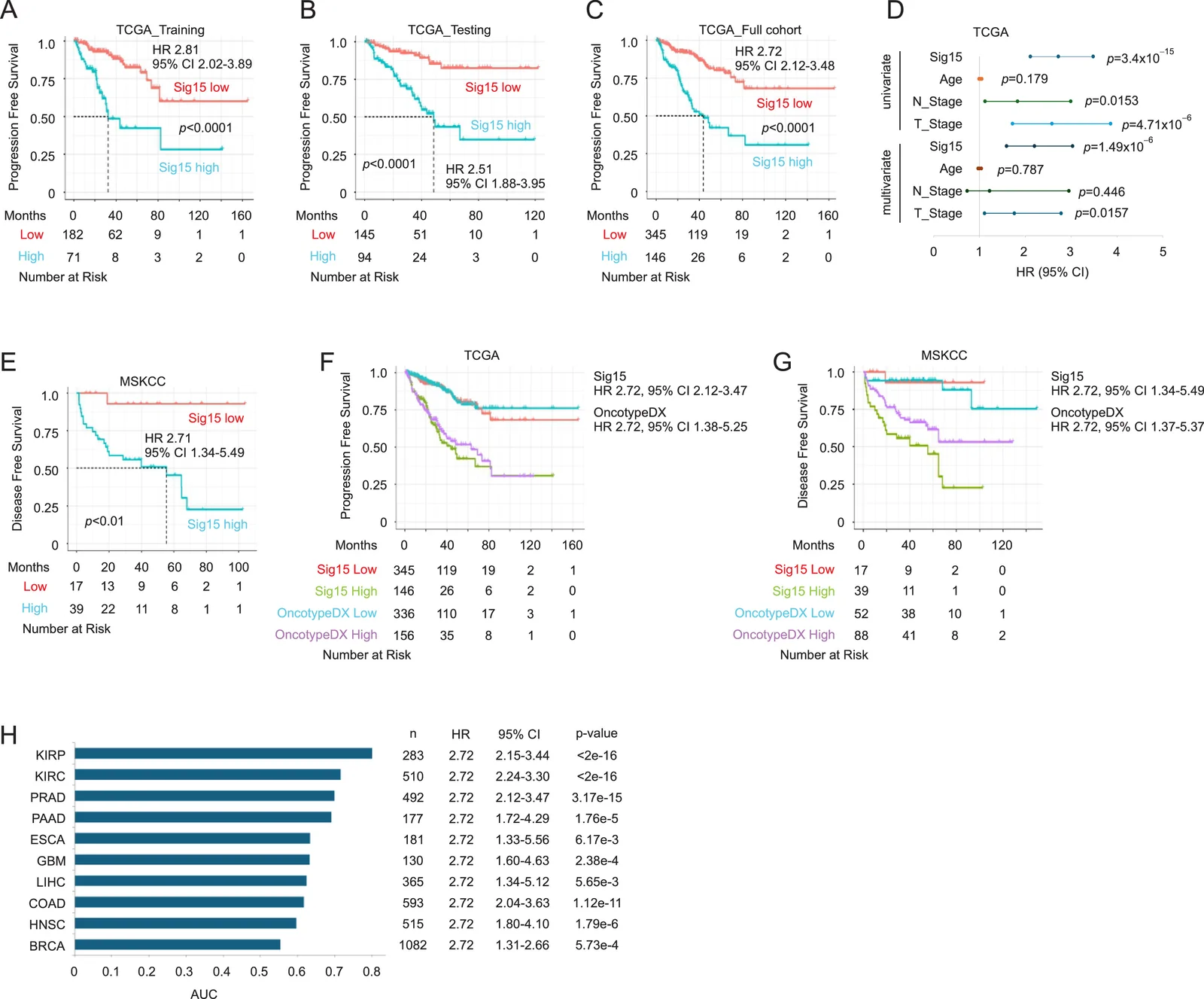
Sig15 predicts efficiently PC risk of recurrence. (A) Coefficients (Coefs) of Sig15 component genes were derived from the TCGA Training sub-dataset (n = 263) and used to compute the Sig15 risk score. (B, C) Sig15 risk scores for tumors in the Testing and full datasets were calculated using the coefs produced from the Training sub-dataset and used to construct the respective survival curves. The hazard ratio (HR) and 95% CI are presented. (D) Univariate and multivariate Cox analysis for Sig15, age at diagnosis, N-stage (N0 Vs N1) and T-stage (T2 Vs T3) in the TCGA cohort. For each variable, HR, 95% CI and associated p-value are presented. Analysis was performed using the R survival package. (E) Kaplan-Meier survival curve for disease-free survival in the MSKCC PC cohort (n = 140) was constructed using cohort-specific coefficients. (F, G) Stratification of PC recurrence risk in the indicated PC cohort using the risk score of Sig15 and Oncotype DX. (H) ROC AUC values of Sig15 in discriminating the prognostic outcome in the indicated cancer type. HR, 95% CI and p-value were derived using the survival package in R. BRCA: breast invasive carcinoma; COAD: colon adenocarcinoma; ESCA: esophageal carcinoma; GBM: Glioblastoma Multiforme; HNSC: head and neck squamous cell carcinoma; KIRC: Kidney renal clear cell carcinoma; KIRP: Kidney papillary cell carcinoma; LIHC: liver hepatocellular carcinoma; PAAD: pancreatic adenocarcinoma; and PRAD: Prostate adenocarcinoma.

We further validated Sig15 in an independent PC cohort (MSKCC) using cohort-specific coefficients, i.e. via computing the respective coefficients using the MSKCC dataset (Fig. 4E). Oncotype DX is the most well-characterized and established multigene panel assessing PC recurrence risk [31,32]. Benchmarked against Oncotype DX, Sig15 exhibits comparable prediction of PC recurrence in both TCGA and MSKCC datasets (Fig. 4F, G). We performed an additional validation by testing Sig15-derived prediction of poor prognosis across 10 cancer types. Sig15 significantly stratified poor overall survival (OS) with an AUC>0.55 in nine cancer types (Fig. 4H). Collectively, these findings provide comprehensive validation for Sig15 as a novel multigene biomarker for PC.

### 3.5 Characterization of Sig15

The novelty of Sig15 is reflected by 11 of its 15 genes with no prior knowledge in PC (Supplementary Table 5). The remaining four genes (*AMH, GOLGA8B, ARHGAP33* and *ANKLE1*) have been reported in PC (Supplementary Table 5). Knockdown of GOLGA8B sensitizes CRPC cells to Cabazitaxel, a chemotherapeutic agent [33]. ARHGAP33 is a potential risk factor in PC [34], and ANKLE1 is associated with poor prognosis [35]. AMH, a protein involved in male sex differentiation, displays correlations with BCR and advanced disease stage [36].

We determined the expression status of each component gene in PC. Among the 15 genes, 12 genes were significantly upregulated (Supplementary Fig. 4). Notably, six murine orthologs of Sig15 were significantly upregulated (*p* < 0.001) in TRAMP-C2 LINC01089 tumors, including *Ankle1, Col11a2, Miox, Slc9a5, Arhgap33* and *Ly6g5b* (Fig. 5A). Among these 6 genes, ANKLE1, COL11A2, SLC9A5, ARHGAP33, and LY6G5B were associated with biochemical recurrence (Fig. 5B-F) and three (MIOX, SLC9A5, and ARHGAP33) were upregulated in metastasis (Fig. 5G-I). Of note, COL11A2, SLC9A5, LY6G5B, and MIOX remain without prior knowledge in PC (Supplementary Fig. 4). Collectively, the above evidence supports Sig15 biomarker potential and suggests its relevance to LINC01089-promoted PC tumorigenesis.

**Fig. 5:**
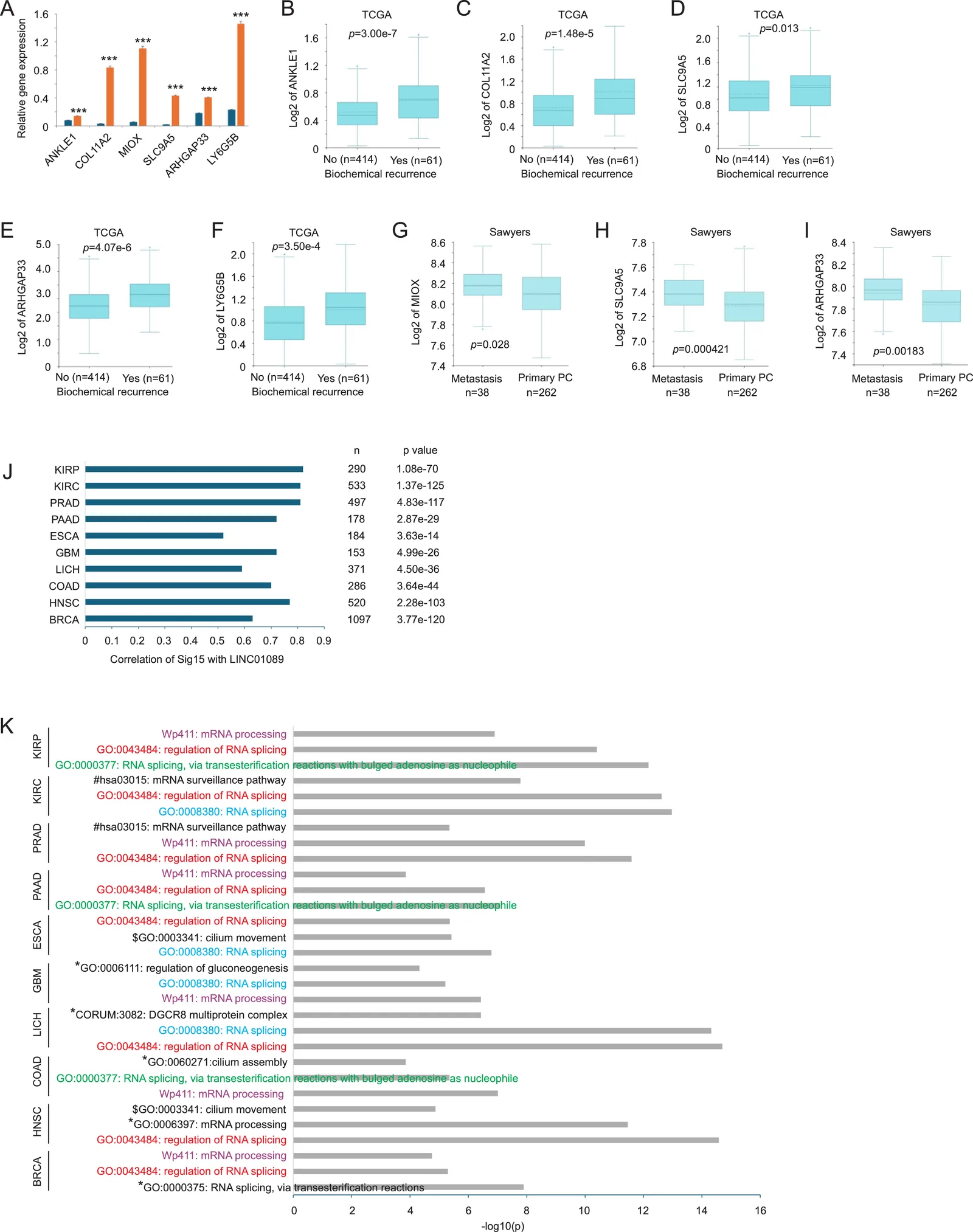
Characterization of Sig15. (A) Relative gene expression of the indicated Sig15 component murine genes was determined using real-time qPCR in TRAMP-C2 EV (n = 6) and TRAMP-C2 LINC01089 (n = 6) tumors. The 2^−ΔCt^ method was used to calculate changes in gene expression, which were normalized to actin. Quantifications are presented as mean±SD. *** *p* < 0.001 by 2-tailed Student *t*-test. (B-F) Expression of the indicated Sig15 component genes in primary PC without and with progression to biochemical recurrence in the TCGA (n = 475) dataset. (G-I) Expression of MIOX, SLC9A5, and ARHGAP33 in primary and metastatic PCs in the Sawyers (n = 300) dataset. (J) Pearson correlation of Sig15 metagene expression and LINC01089 expression in the indicated TCGA pan-cancer dataset. Analyses were performed using R2: Genomics Analysis and Visualization Platform (http://r2.amc.nlhttp://r2platform.com). Statistical analyses were provided by the R2 Platform. (K) Genes positively correlated with Sig15 were obtained from KIRP (R ≥ 0.7, *p* < 0.01), KIRC (R ≥ 0.7, *p* < 0.01), PRAD (R ≥ 0.6, *p* < 0.01), ESCA (R ≥ 0.5, *p* < 0.01), GBM (R ≥ 0.65, *p* < 0.01), LIHC (R ≥ 0.5, *p* < 0.01), COAD (R ≥ 0.6, *p* < 0.01), HNSC (R ≥ 0.5, *p* < 0.01), and BRCA (R ≥ 0.5, *p* < 0.01). Different correlation coefficients (R) were chosen to ensure around 300 positively correlated genes. Pathway enrichment analyses were performed using Metascape (https://metascape.org/gp/index.html#/main/step1). The top 3 enriched pathways are presented. Commonly enriched pathways across cancer types are colored or marked with # or $. *: pathways enriched uniquely in the indicated cancer types.

We further observed high-level (R > 0.5) correlations between LINC01089 and Sig15 across 10 cancer types (Fig. 5J); in these cancer types, Sig15 stratifies poor recurrence (PRAD) and inferior OS (Fig. 4H). These correlations support the derivation of Sig15 from the LINC01089-associated DEGs in PC and validate the general relationship between Sig15 and the LINC01089 network across multiple cancer types. Given that the LINC01089 network was enriched in RNA splicing in PC (Fig. 3), we reasoned that enrichments of RNA splicing and related events would occur in Sig15 across these 10 cancer types. To examine this possibility, we derived genes positively correlated with Sig15 (R ≥ 0.5, *p* < 0.01) from the 10 cancer types and demonstrated that the top three enriched pathways were centered on RNA splicing and related processes (Fig. 5K). Furthermore, these enrichments were highly conserved, evidenced by GO:0008,389: RNA splicing, GO:0043,484: regulation of RNA splicing, and Wp411: mRNA processing being commonly present across these cancer types (Fig. 5K), consistent with GO:0008,389: RNA splicing as the dominant enriched event in LINC01089-related DEGs (Fig. 3A). Collectively, these observations suggest that Sig15 stratifies poor prognosis across these cancer types likely by capturing the RNA splicing abnormalities; an established driver of cancer progression [29,37,38].

### 3.6 LINC01089 as a potential liquid-based biomarker

We further studied the potential of LINC01089 as a liquid biomarker. This possibility is supported by LINC01089 expression in basal and luminal epithelial cells of normal human prostate (Supplementary Fig. 5A). As a well-established urine biomarker of PC [20,39], PCA3 is minimally detected in basal epithelial cells (Supplementary Fig. 5B). We obtained 235 plasma samples from the Ontario Tumor Bank (OTB) for patients diagnosed with primary PC prior to treatment (Table 1) and 24 plasma samples from healthy individuals from the American Association for Clinical Chemistry (AACC) who were screened for multiple chronic diseases (diabetes, high blood pressure, renal disease, heart disease, deep vein thrombosis), cancer or undergoing treatments at the time of collection. Hemolysis assessment performed on plasma reported an A414/375 index between 0.160 and 0.203 (good to acceptable threshold) [40].

**Table 1:** Clinico-pathological characteristics of plasma donors.

Prostate Cancer Plasma donors⁎	
Variable	N (%); Mean (Min-Max)
Age	
<70:	192 (81.7%)
>70:	43 (18.3%)
Grade Group	
G1	50 (21.3%)
G2	50 (21.3%)
G3	50 (21.3%)
G4	35 (14.9)
G5	50 (21.3%)
PSA (µg/L)	9.29 (1.10–73.01)
Progression Status	
None	166 (70.6%)
Recurred	16 (6.8%)
N/A	53 (22.6%)
Nodal Metastasis	
Yes	7 (3.0%)
No	215 (91.5%)
N/A	13 (5.5%)
Control Individuals Plasma donors⁎⁎	
Variable	N (%); Mean (Min-Max)
Age	61 (51–74)
Questionary:	
1. Do you have renal disease?	
No	24 (100%)
2. Do you have High Blood Pressure?	
No	24 (100%)
3. Do you have any heart Condition?	
No	24 (100%)
4. Do you have a history of heart disease?	
No	24 (100%)
5. Are you currently being treated for cancer or do you have a history of cancer within the last 5 years?	
No	24 (100%)
6. Are you diabetic?	
No	24 100%)
7. Are you a current Smoker or a former smoker?	
Current	4 (16.6%)
Former	20 (83.4%)
8. Have you ever been told you have high cholesterol?	
Yes	2 (8.3%)
No	22 (91.7%)
9. Do you consider yourself to be generally healthy	
Yes	24 (100%)
10. Do you exercise regularly?	
Yes	22 (91.7%)
No	2 (8.3%)

⁎ Plasma Samples obtained from the Ontario Tumour Bank (OTB), diagnosed with primary Prostate adenocarcinoma.

⁎⁎ Plasma Samples obtained from the American Association for Clinical Chemistry (AACC).

Elevations of LINC01089 in patients’ plasma were demonstrated (Fig. 6A), which is also positively associated with Grade Group (Fig. 6B). As expected, plasma PCA3 is increased in PC (Fig. 6C), showing a significant positive correlation with LINC01089 (Fig. 6D). Plasma LINC01089 exhibited a significant correlation with the PSA score (Fig. 6E). Plasma PCA3 also significantly correlated with PSA (Fig. 6F) at a comparable level to LINC01089 (comparing Fig. 6E to F). These observations provide strong validation for the association of plasma LINC01089 with PC. Nonetheless, the modest correlation between LINC01089 and PSA may reflect the non-specific status of PSA in diagnosis; in this regard, LINC01089 might be explored to complement PSA-based PC diagnosis (see Discussion for details).

**Fig. 6:**
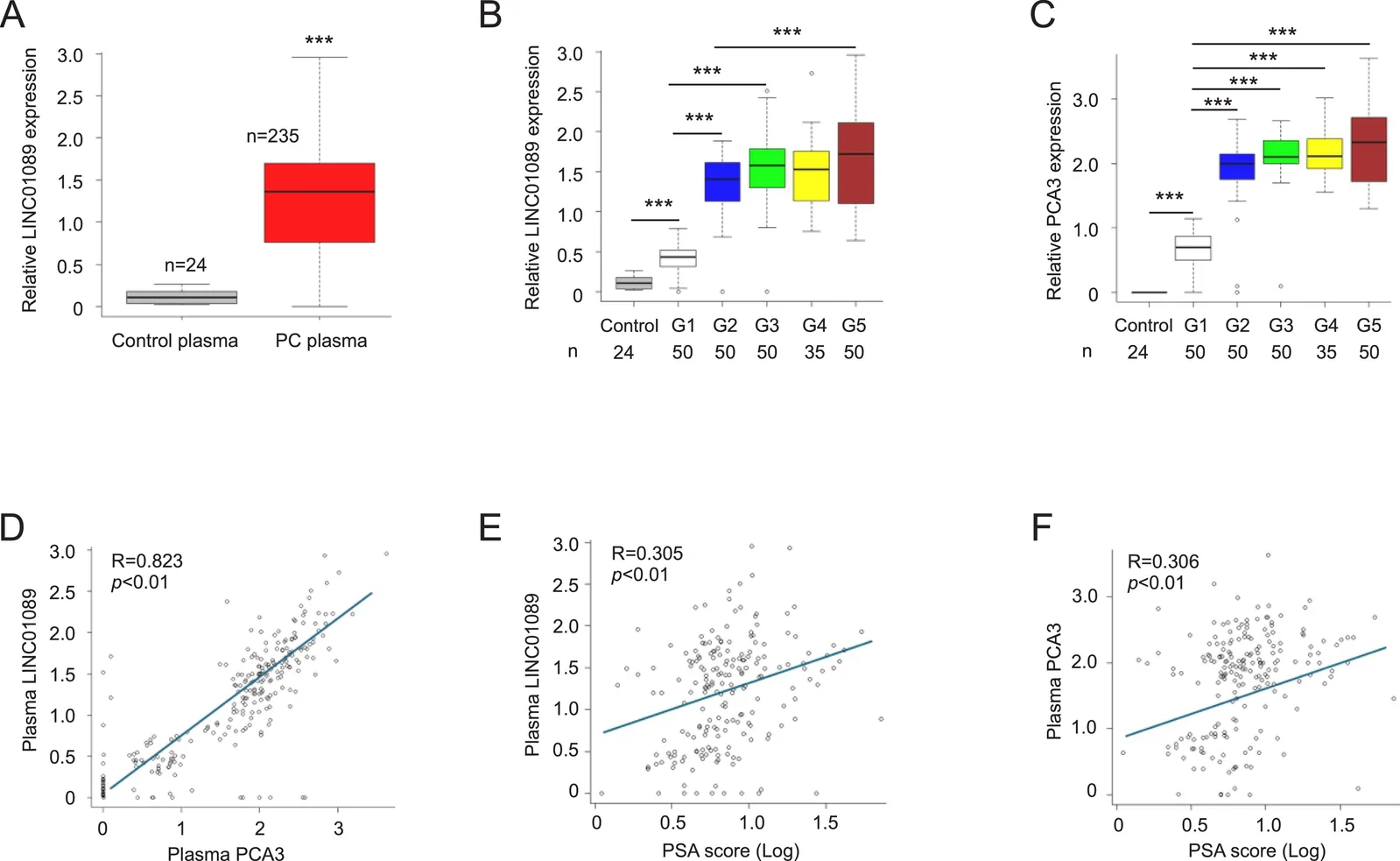
LINC1089 as a potential liquid-based biomarker in PC. (A-C) Total RNA from patients' plasma diagnosed with primary PC (n = 235) and control individuals (n = 24) was extracted using TRIzol. Real-time qPCR was utilized to determine LINC01089 relative expression between PC and control (A) and among PC grade groups (B), as well as relative PCA3 expression among PC grade groups (C). The 2^−ΔCt^ method was used to calculate changes in gene expression, which were normalized to actin. Statistical analysis was performed using one-way ANOVA followed by a Tukey-HSD post-hoc test. (D) Pearson correlation analysis between LINC01089 and PCA3 expression. The correlation coefficient and p-value are shown. (E, F) Pearson correlation analysis between LINC01089 and PSA score (E), and PCA3 and PSA score (F). The correlation coefficient and p-value are presented.

## 4 Discussion

Improvement in management of PC requires advances in diagnosis, risk stratification, and reducing resistance to ADT. This research contributes to the current understanding of these overarching issues.

PSA screening remains a major step in PC diagnosis [39,41]. However, the screen is well-recognized for being non-specific and leading to unnecessary biopsies [42]. The inclusion of the FDA-approved urinary PC biomarker PCA3 together with PSA improved the diagnostic issue, particularly for cases with elevated PSA and a negative biopsy [20,43]. The urine PCA3 test (Progensa), at a cutoff point of 35, differentiates PC from benign with a specificity of approximately 56%; however, its sensitivity is considerably reduced in patients with PSA ≤ 3ng/ml or Gleason score ≤7, and it failed to detect clinically aggressive PC [44]. Moreover, the inconsistency in PCA3 association with PC relevant pathological characteristics in different studies/cohorts restricted its diagnostic applications [44,45].

Our research suggests that plasma LINC01089 may improve PC diagnosis. PSA (prostate- specific antigen) is a classic AR target, and its level is influenced by changes in AR activity affected by multiple non-oncogenic conditions. PCA3 (prostate cancer antigen 3) is androgen- responsive and interacts with AR signaling, which may limit PCA3 biomarker value in PC diagnosis. LINC01089 is not an AR target, as its level was not reduced in LNCaP cells cultured in androgen-depleted media (Fig. 2C). Nonetheless, LINC01089 offers LNCaP and TRAMP-C2 cells resistance to androgen withdrawal (Fig. 2D, E). It is thus tempting to propose that combining LINC01089 with PSA and/or PCA3 may enhance PC diagnosis. Given the advent of prostate-specific membrane antigen (PSMA)-based imaging in PC diagnosis with 68Ga-PSMA-11 being approved by the FDA [46], exploration of plasma LINC01089 with 68Ga-PSMA-11 imaging can also be investigated in the future to improve PC diagnosis.

LINC01089 network (Overlap3 and Sig15) displays prognostic biomarker value. Both multigene panels reflect the RNA splicing properties of cancer, a major contributor to alterations in gene expression, which lies at the heart of cancer progression [37,38]. This biology might aid Sig15 in the stratification of the recurrence risk of PC and poor prognosis across the other nine cancer types. While the properties of proliferation, immunity, and communication between tumor cells and stromal cells have been widely explored to assess PC progression [2,31,47], RNA splicing has not been extensively explored for its application [29]. Nonetheless, several alternative splicing events and/or factors have been identified as crucial for PC progression [7,29,48]. Furthermore, Sig15 exhibits biomarker potential comparable to Oncotype DX, the most extensively investigated multigene panel for PC [31]. Collectively, our study may contribute to the stratification of PC recurrence risk. Realization of this potential will require both retrospective and prospective studies using large cohorts.

LINC01089 and its network (Overlap3 and Sig15) biomarker potentials are further supported by its function in promoting PC. Overexpression of LINC01089 in human and murine PC cells promoted multiple oncogenic activities in vitro and TRAMP-C2 cell-based tumor growth in syngeneic mice. Importantly, all component genes of Overlap3 and 6 of the 15 component genes of Sig15 were significantly upregulated in TRAMP-C2 LINC01089 tumors over EV tumors, supporting the relevance of Overlap3 and Sig15 to LINC01089-caused PC growth. While the detailed mechanisms underlying LINC01089-enhanced PC are not clear, our research suggests that LINC01089 facilitates AR signaling activation, evidenced by increased AR expression and nuclear accumulation. As a result, LINC01089 conferred resistance to androgen deprivation in LNCaP and TRAMP-C2 cells. Nonetheless, the mechanism by which LINC01089 facilitates AR signaling and enhances PC growth should be further investigated.

Our evidence also suggests that the oncogenic role of LINC01089 in PC could be related to dysregulation of RNA splicing, thus producing an aberrant transcriptome [29], which might be a mechanism for LINC01089-derived oncogenic actions. Notably, LINC01089 was reported to play a role in alternative RNA splicing in hepatocellular cancer [16]. Nonetheless, we did not directly investigate LINC01089’s role in regulating RNA splicing, which is a limitation of this study. Given the importance of RNA splicing dysregulation in PC, the involvement of LINC01089 in RNA splicing in PC should be examined in the future.

Our study has additional limitations. First, we focus on the LINC01089 overexpression model in PC. This approach was based on 1) the heterogenous expression of LINC01089 in primary PC (Fig. 1D, E), 2) PCs with elevated LINC01089 expression displayed aggressive clinical features (Fig. 1F-H), 3) among the three common human PC lines (DU145, PC3, and LNCaP), LNCaP cells expressed the lowest level of endogenous LINC01089 (Fig. 1A), 4) LINC01089 overexpression in all three PC lines enhanced multiple oncogenesis-relevant processes in vitro (Fig. 1I-Q), 5) both LNCaP LINC01089 and TRAMP-C2 LINC01089 cells exhibited resistance to androgen withdrawal in vitro, and 6) TRAMP-C2 LINC01089 cells also exhibited elevated oncogenesis in vivo. Collectively, through analyses using PC cells with different levels of endogenous LINC01089 and involving multiple experimental platforms (in vitro, in vivo, androgen withdrawal, and translational studies), the current study supports the conclusion that LINC01089 facilitates PC. Nonetheless, this concept needs to be strengthened via thorough studies using knockdown and/or knockout approaches in the future. Second, although we demonstrated that LINC01089 possesses a key role in PC progression, the precise mechanism remains unclear.

## Funding

This research was supported by a CIHR grant (202309PJT-506896-C2-CEBA-108276) to DT and a donation from the Masonic Foundation of Ontario to DT.

## CRediT authorship contribution statement

**Sandra Vega Neira:** Writing – review & editing, Writing – original draft, Methodology, Investigation, Formal analysis, Conceptualization. **Damu Tang:** Writing – review & editing, Writing – original draft, Supervision, Funding acquisition, Formal analysis, Conceptualization.

## Declaration of competing interest

The authors declare that they have no known competing financial interests or personal relationships that could have appeared to influence the work reported in this paper.

## Data Availability

Data will be made available on request.
