# Supplementary material for: The long non-coding RNA LINC01089 facilitates prostate cancer progression
Source: BBA Adv. 2026 Oct 14;10:100205. doi: 10.1016/j.bbadva.2026.100205 (PMC13696824; doi:10.1016/j.bbadva.2026.100205)
Supplement: Supplementary file 1 [file mmc1.pdf]

Supplementary Figure 1

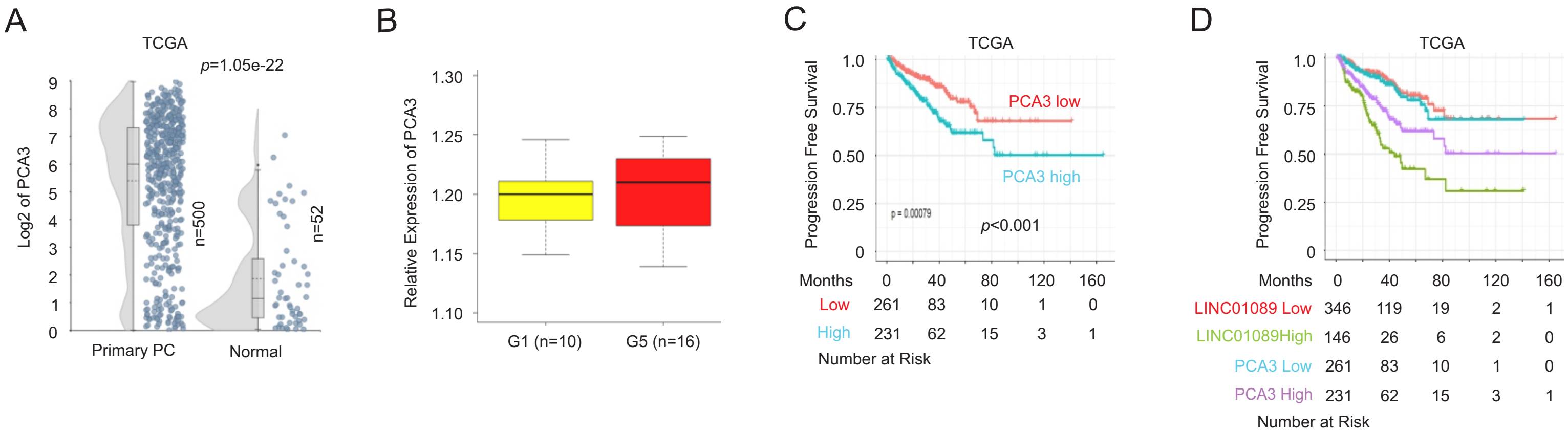

Supplementary Figure 1. PCA3 expression in PC. (A) PCA3 expression (mRNA) in primary PC (n=500) and normal tissue (n=52) in the TCGA dataset. Analysis was performed using R2: Genomics Analysis and Visualization Platform (http://r2.amc.nl http://r2platform.com). (B) mRNA expression quantification by real-time qPCR of PCA3 between Group grade 1 (n=10, Gleason score 5-6) and grade 5 (n=16, Gleason score 9-10). The 2−ΔCt method was used to calculate changes in gene expression and normalized to actin. Quantifications were presented as mean ± SD (Standard Deviation). Kaplan-Meier progression-free survival (PFS) curve of (C) PCA3 and (D) its comparison to LINC01089 in the TCGA cohort. Cut-off points were estimated using Maxstat, and log-rank test analysis were carried out with the R survival package.

Supplementary Figure 2

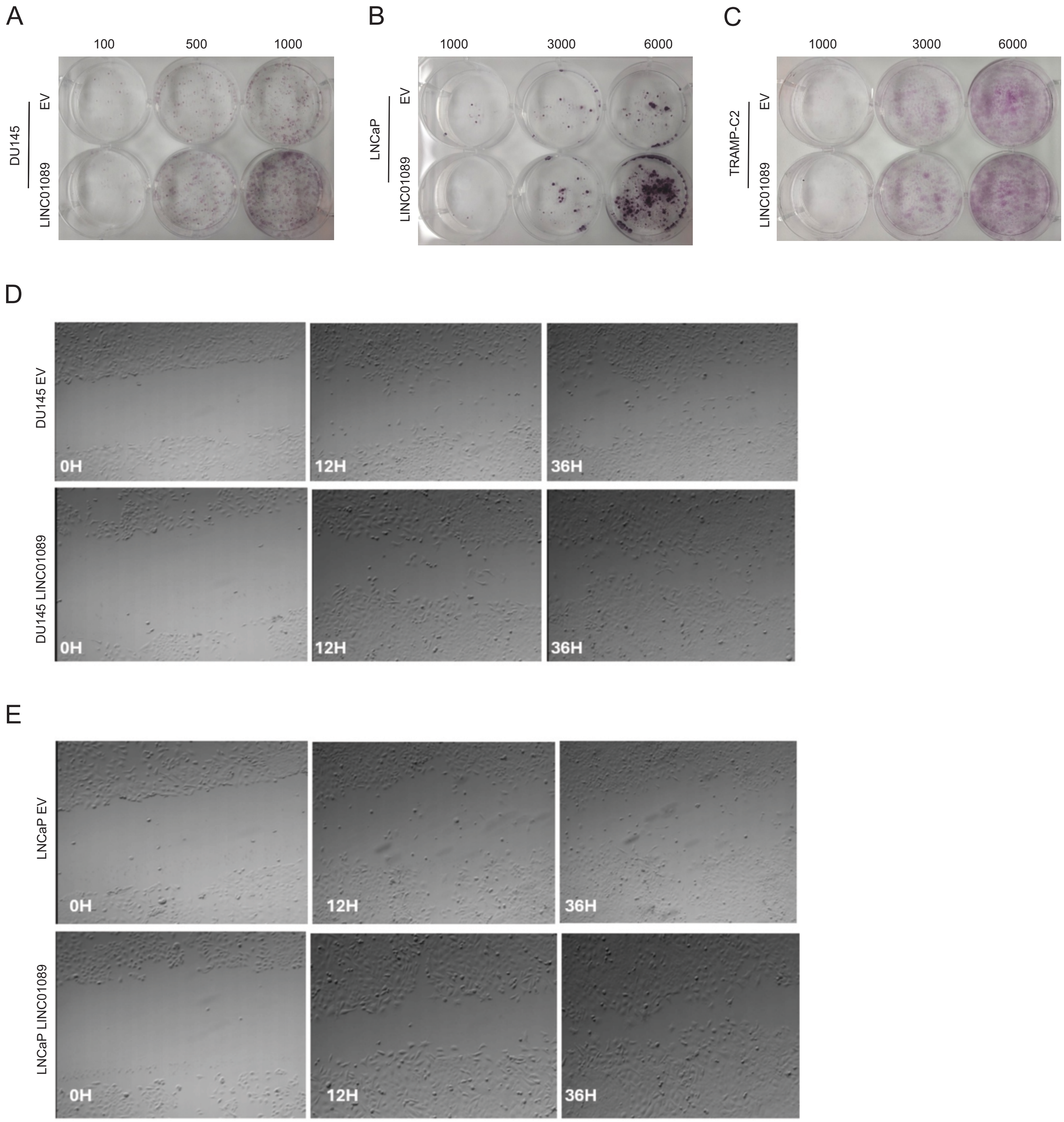

Supplementary Figure 2. LINC01089 facilitates PC cell forming colony and migration. (A, B, C) The indicated EV and LINC01089 stable lines were seeded in 6-well plates at the indicated density/well and cultured for 8 days till colonies formed. Cells were stained by 0.5% crystal violet. Typical images are presented. (D, E) The indicated stable lines were seeded in a 6-well plate until reaching 90% confluency. A horizontal scratch was made on each well using a pipette tip, and migration was monitored every 6 hours. Images were taken under the light microscope, and the wound width (mm) was measured using ImageJ software. Typical images are presented at the indicated time point. All experiments were repeated three times.

Supplementary Figure 3

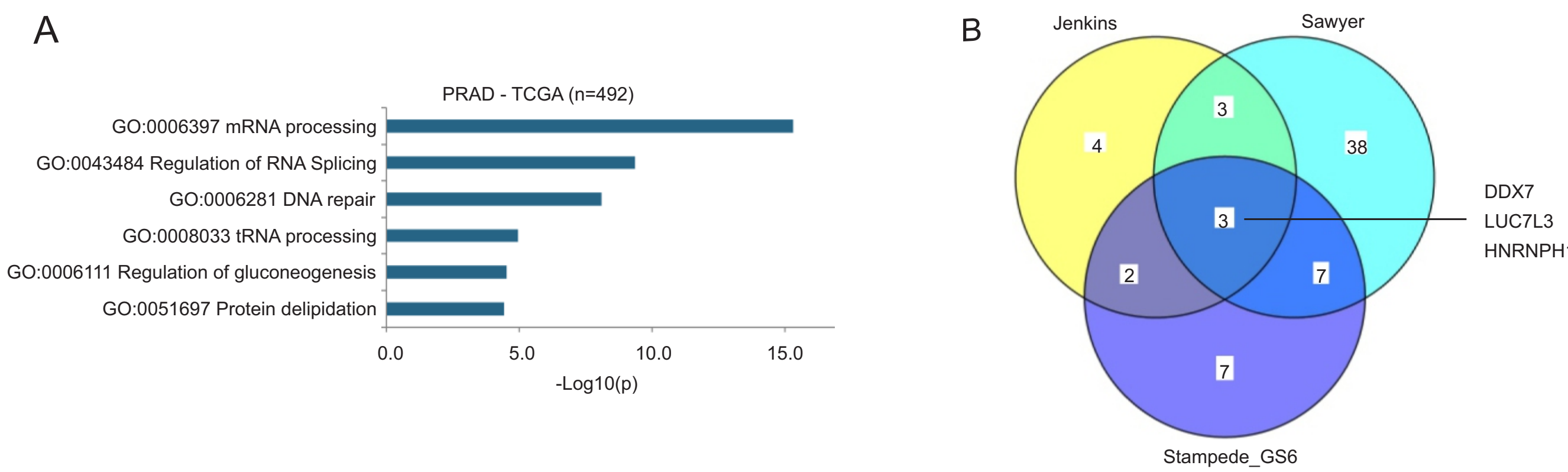

Supplementary Figure 3. Characterization of LINC01089 correlated genes. (A) Genes positively correlated with LINC01089 expression (R⩾0.55, p<0.0001) were derived from the TCGA PC dataset, followed by enrichment analysis using Metascape (https://metascape.org/gp/index.html#/main/step1). (B) LINC01089 positively correlated genes were obtained from Jenkins (primary PC, n=333; R⩾0.5; p<0.05), Sawyers (metastasis, n=38; R⩾0.7; p<0.05), and Stampede (GS6-7 PC, n=348; R⩾0.5; p<0.05) dataset. The RNA slicing pathway was a top-enriched pathways, which were analyzed with Venn diagram. The three shared (Overlap3) RNA splicing factors are indicated.

Supplementary Figure 4

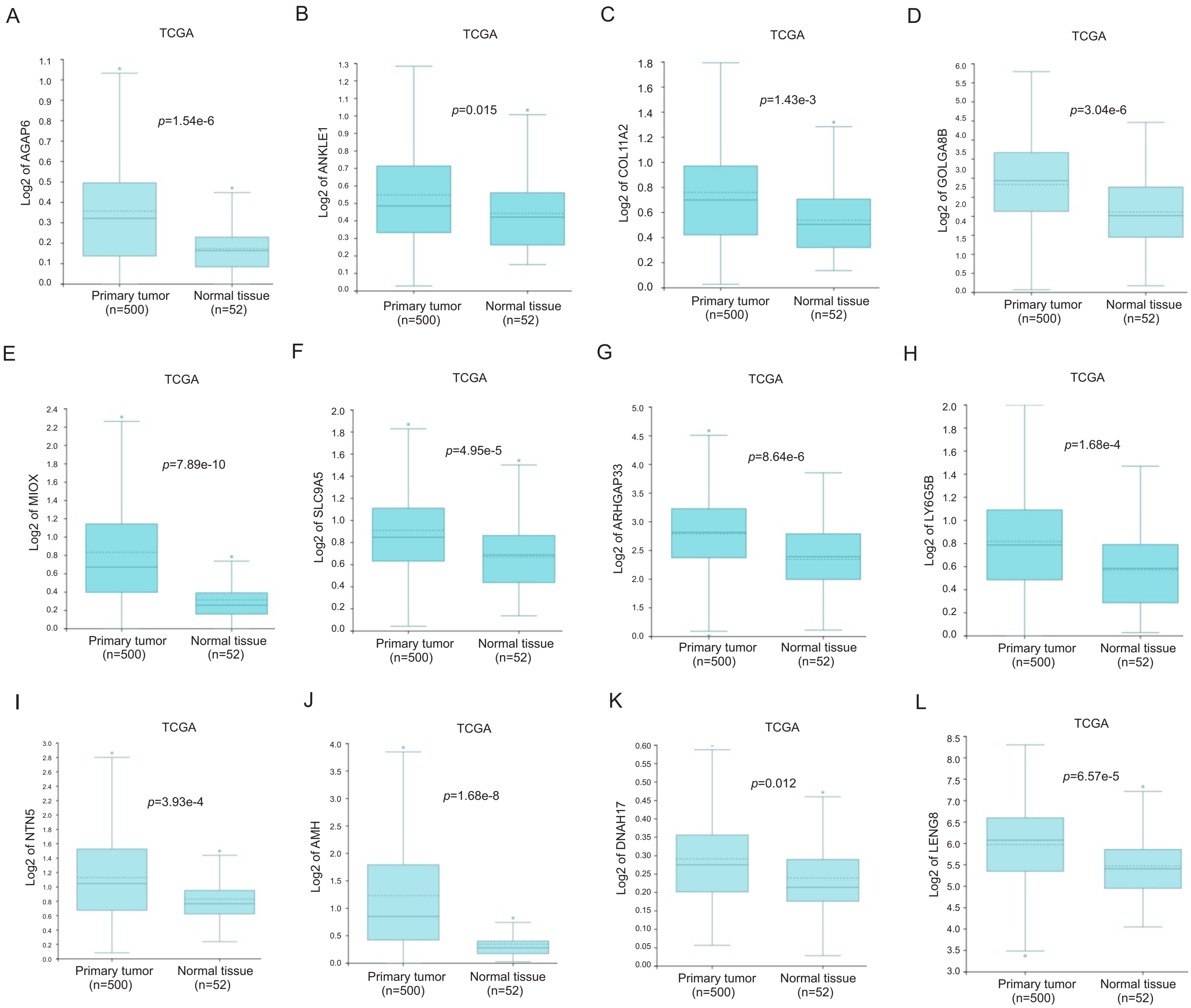

Supplementary Figure 4. Sig15 component gene expression in PC vs normal prostate tissues. Analyses were performed using the TCGA dataset in R2 Genomics Analysis and Visualization Platform (http://r2.amc.nl http://r2platform.com). The expression of the indicated genes was presented as Log2 transformed data. Statistical analyses were performed by the R2 Platform using one-way ANOVA.

Supplementary Figure 5

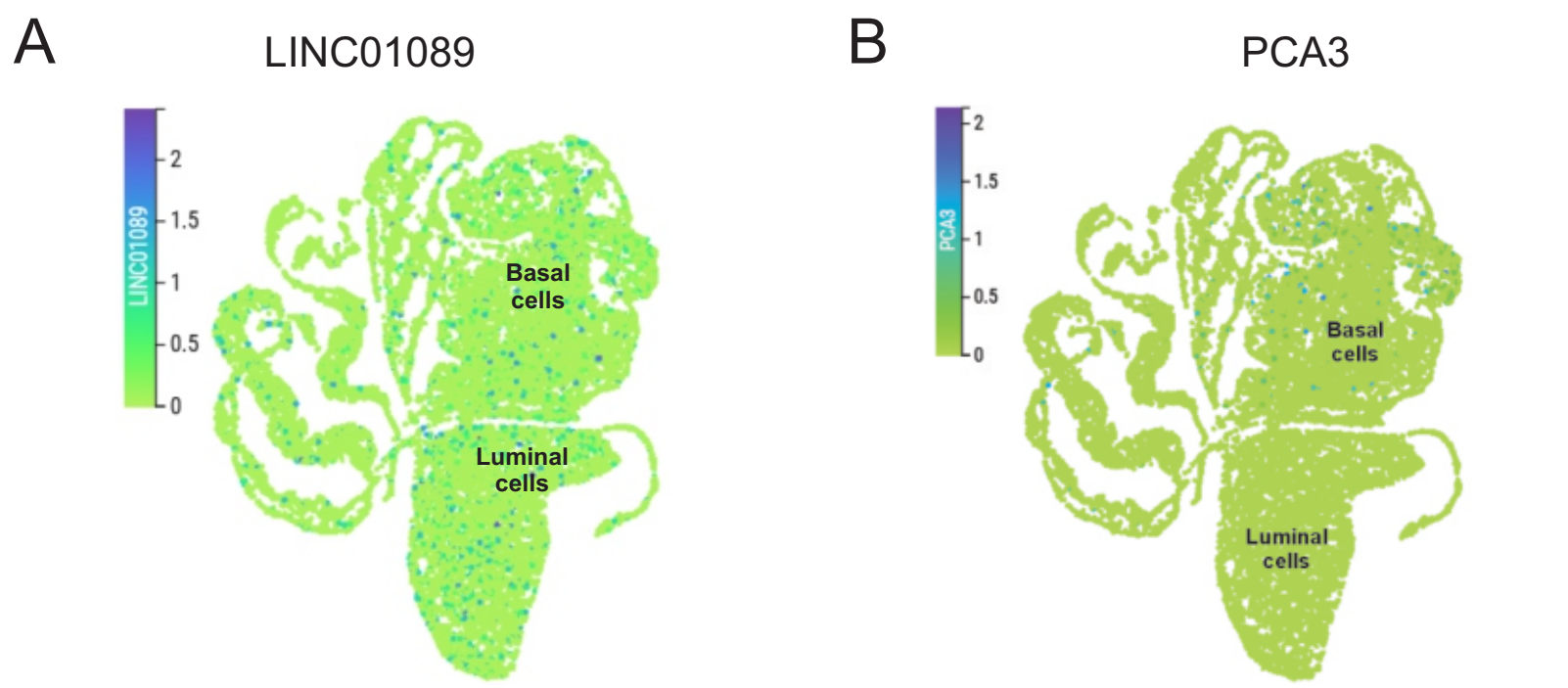

Supplementary Figure 5. Expression of LINC01089 and PCA3 at the single cell level in human prostate. LINC01089 (A) and PCA3 (B) expression in single cell of human prostate were analyzed using the CZ CELLxGENE discovery platform. Epithelial cell subtype population is indicated.
